# The Path from Nasal Tissue to Nasal Mucosa on Chip: Part 1—Establishing a Nasal In Vitro Model for Drug Delivery Testing Based on a Novel Cell Line

**DOI:** 10.3390/pharmaceutics15092245

**Published:** 2023-08-30

**Authors:** Sebastian Bendas, Eugen Viktor Koch, Kristina Nehlsen, Tobias May, Andreas Dietzel, Stephan Reichl

**Affiliations:** 1 Institute of Pharmaceutical Technology and Biopharmaceutics, Technische Universität Braunschweig, Mendelssohnstraße 1, 38106 Braunschweig, Germany; s.bendas@tu-braunschweig.de; 2 Center of Pharmaceutical Engineering, Technische Universität Braunschweig, Franz-Liszt-Straße 35 a, 38106 Braunschweig, Germany; eu.koch@tu-braunschweig.de (E.V.K.); a.dietzel@tu-braunschweig.de (A.D.); 3 Institute of Microtechnology, Technische Universität Braunschweig, Alte Salzdahlumer Straße 203, 38124 Braunschweig, Germany; 4 InSCREENeX GmbH, Inhoffenstraße 7, 38124 Braunschweig, Germany; kristina.nehlsen@inscreenex.com (K.N.); tobias.may@inscreenex.com (T.M.)

**Keywords:** nasal epithelial cell line, nasal mucosa, in vitro model, goblet cells, mucus, ciliated cells, TEER measurement, cell layer capacitance, drug permeation, MucilAir™

## Abstract

In recent years, there has been a significant increase in the registration of drugs for nasal application with systemic effects. Previous preclinical in vitro test systems for transmucosal drug absorption studies have mostly been based on primary cells or on tumor cell lines such as RPMI 2650, but both approaches have disadvantages. Therefore, the aim of this study was to establish and characterize a novel immortalized nasal epithelial cell line as the basis for an improved 3D cell culture model of the nasal mucosa. First, porcine primary cells were isolated and transfected. The P1 cell line obtained from this process was characterized in terms of its expression of tissue-specific properties, namely, mucus expression, cilia formation, and epithelial barrier formation. Using air–liquid interface cultivation, it was possible to achieve both high mucus formation and the development of functional cilia. Epithelial integrity was expressed as both transepithelial electrical resistance and mucosal permeability, which was determined for sodium fluorescein, rhodamine B, and FITC-dextran 4000. We noted a high comparability of the novel cell culture model with native excised nasal mucosa in terms of these measures. Thus, this novel cell line seems to offer a promising approach for developing 3D nasal mucosa tissues that exhibit favorable characteristics to be used as an in vitro system for testing drug delivery systems.

## 1. Introduction

In recent years, an increase has been observed in drug candidates with reduced peroral bioavailability due to poor water solubility, low stability in the gastrointestinal tract, low intestinal absorption, or an increased first pass effect. Thus, alternative routes of administration for these active ingredients must be considered; among these, a promising route is the application of drugs via the nasal mucosa. From a biopharmaceutical point of view, the combination of the strong vascularization of nasal tissue and the barrier properties of the nasal epithelium, which are lower than those of the intestinal epithelium, favors the rapid removal of the applied drug and, thus, the rapid achievement of therapeutic drug levels in the blood. These favorable properties are exploited, for example, in nasally applied fentanyl for the treatment of severe breakthrough pain [1,2]. In addition, due to the poor expression of metabolizing enzymes in the nasal mucosa [3], the first-pass effect can be lowered compared with oral administration [4]. The weaker paracellular barrier between nasal epithelial cells and the considerably lower proteolytic conditions compared to the gastrointestinal tract make peptide hormones such as calcitonin, desmopressin, and oxytocin suitable for nasal administration [5]. In contrast, the epimucosal mucus layer, due to its high viscosity, and the strong activity of cilia-forming mucociliary clearance (MC), which strongly limit the residence time of nasal formulations, lower bioavailability [6]. Due to the complexity of the nasal mucosa, studies investigating the pharmacokinetic properties of nasal drug application are almost exclusively performed in animals using in vivo models and/or ex vivo tissue specimens. However, organotypic in vitro test systems are needed to reduce the number of animal tests required in line with the “3Rs principle”. Current in vitro test systems mainly consist of isolated human nasal epithelial cells or the RPMI 2650 cell line, but neither model can meet all of the relevant requirements. The advantages for the use of primary cultured human nasal epithelium are the presence of all physiological cell types, the organotypic structure, and similar protein expression patterns [7,8]. The drawbacks of primary nasal epithelial cell cultures are the limited availability of cell material, especially of human origin, limited lifespan, low subcultivation rate of the cells, and pronounced interindividual variation depending on the donor that also affects the formation of barrier properties of the cell culture models [9]. While human epithelial cells of the nasal mucosa in vivo typically have transepithelial electrical resistance (TEER) values of circa 100 Ω cm^2^ [10,11], primary cultures mostly show values that are significantly higher (>1000 Ω cm^2^) [12,13]. However, organotypic properties (expression of mucus and active cilia) can be increased by culturing at the air–liquid interface (ALI) [9]. Commercially available models that are based on primary cultures already exist. For example, MucilAir™ is a well-established model developed by Epithelix consisting of human nasal epithelial cells at low passage on Transwell^®^ inserts. Furthermore, the human cell line RPMI 2650 derived from a carcinoma of the nasal septum and commonly used as an in vitro model of nasal epithelium [14] is characterized by organotypic TEER values when cultured at the air–liquid interface [15]. In addition, the permeability for several marker substances [16] as well as the expression patterns of ABC (ATP binding cassette) and SLC (solute carrier) transporters are very similar to those of excised human tissue [17,18,19]. Compared to primary cell cultures, this cell line is characterized by easy cultivation as well as unlimited availability, allowing experiments to be performed on a large scale. However, the disadvantages are the tumor-like proliferation of these cells and their anomalous histology, which occurs due to smaller cell size and multilayer growth. In previous studies, no ciliated epithelial cells were detected [20]; moreover, the expression of mucus was also negligible [21,22]. However, in recent years, it has been shown that the cultivation of RPMI 2650 under dynamic conditions in which air flow is applied has a positive effect on the expression of mucus [23,24]. The same observation was made by Even-Tzur et al., who cultured human nasal primary cells under air flow at the ALI [25,26]. This indicates that the cultivation of established in vitro models under dynamic conditions in organ-on-a-chip systems can help to further increase organotypic qualities and will become even more important in the coming years.

Since both human primary cells and RPMI 2650 cells do not represent a fully satisfactory model of the nasal mucosa, the aim of this project was to develop an organotypic and dynamic in vitro test system based on a new immortalized nasal epithelial cell line that mimics mucociliary clearance properties. The first step in achieving this goal was the development of an isolation protocol for the selective generation of epithelial cells from native tissue derived from pig snouts; this served as a starting point for the subsequent application of the method to establish a line of human nasal epithelial cells. Isolated epithelial cells were used to obtain new immortalized cell lines via lentiviral transduction using CI-SCREEN technology [27]. The resulting P1 cell line was then analyzed for the expression of cell type-specific markers and used to develop a static 3D model of the nasal mucosa at the ALI and using LCC (liquid-covered culture). This new air nasal mucosa (ANaMuc) model cultivated under the ALI was examined with respect to the production of mucus, the development of cilia, and the formation of an in vivo-like barrier with typical TEER measurements. The obtained results were compared with 3D models cultured exclusively under submerged conditions (liquid nasal mucosa; LiNaMuc). Additionally, the ANaMuc model was compared with the established MucilAir™ model with respect to the permeation properties of three different marker substances.

## 2. Materials and Methods

### 2.1. Primary Cells

Primary cells were obtained from halved pig snouts provided by a regional slaughterhouse. The specimens were cooled with ice and used for experiments within 1.5 h after removal. Samples were first washed in running tap water, followed by decontamination with 70% ethanol (*v*/*v*). After the removal of the nasal septum, the mucosa was separated from the cartilage tissue using a scalpel. The tissue specimens were washed with phosphate-buffered saline (PBS). The epithelial cells were isolated from the connective tissue by incubating the samples with Accutase^®^ (PAN Biotech, Aidenbach, Germany) for 60 min at room temperature. The epithelial cells were separated using a fine brush (Celletta™ brush cell collector with protective tip, Engelbrecht Medizin- und Labortechnik GmbH, Edermünde, Germany) and resuspended in cell culture medium (HuNASA, InSCREENeX GmbH Braunschweig, Germany). The number of cells was determined using a Vi-CELL XR cell counter (Beckman Coulter, Brea, CA, USA). To improve the adhesion of the isolated cells, cell culture flasks were coated with rat tail collagen solution (1.5 mg/mL). In addition, the isolation of nasal epithelial cells was performed without enzymatic digestion by direct brushing of the nasal mucosa. To characterize the purity of the obtained epithelial primary cultures, immunofluorescence labeling of vimentin was performed to detect contaminating fibroblasts (see Section 2.5 for fluorescence staining procedure). Quantitative evaluation of fluorescence images was performed with ImageJ by counting the structures labeled by anti-vimentin antibodies for each image using the cell counter plugin. In a 2nd step, the respective number of stained cell nuclei was determined. The percentage of fibroblasts was determined by the ratio of vimentin-positive cells to total cell number. For each isolation method, two independent experiments were performed.

### 2.2. Immortalized Cell Lines

After the isolation of porcine primary cells, the cells were cultivated on 12-well plates coated with Epithelial Cell Coating Solution (InSCREENeX GmbH, Braunschweig, Germany). After reaching 80% confluency, the primary cells were transduced with lentiviral vectors encoding the CI-SCREEN gene library [27]. Lentiviral transduction was performed overnight at a multiplicity of infection (MoI) of 5 with 8 µg/mL polybrene (Sigma-Aldrich, Taufkirchen, Germany) supplementation. After 12 h, the culture medium containing lentiviruses was aspirated, and the cells were cultivated for an additional 5 days before selection pressure was applied with 0.2 mg/mL Geneticin (Thermo Fisher, Schwerte, Germany). The transduced cells were placed under selection pressure for 14 days with medium renewal every 4 days, and colonies of proliferating cells became visible. These colonies were surrounded by contaminating fibroblasts. Therefore, the cultures were subjected to sequential splitting, meaning that detachment of the cells was performed with trypsin/EDTA at room temperature, and as soon as the contaminating fibroblasts started to round up, the culture was rigorously washed with PBS. This treatment was repeated once a week for three weeks; finally, the culture consisted entirely of cells displaying an epithelial cell morphology. This culture was expanded without any selection pressure, cryopreserved, thawed, and characterized for cell attachment and viability. At the end of this process, the P1 cell line was selected for further investigation as it displayed a suitable proliferation pattern without any selection pressure, and could be successfully cryopreserved/thawed, thus fulfilling the basic requirements for a novel cell model.

### 2.3. Cell Culture Models

The P1 cell line was seeded on a collagen-coated (see Section 2.1.) 12 mm Transwell^®^ polyester membrane with a 0.4 µm pore size (Costar 3460, Corning, NY, USA) at a density of 0.25 · 10^6^ cells per insert. Cells were submerged (LCC) during culturing for 2 days. There was 1.5 mL basal and 0.5 mL apical HuNASA medium. On day 3 of the culture, the cells were lifted to the ALI after reaching confluence, creating the ANaMuc model. The HuNASA medium on the apical side was removed and the models were supplied with 2 mL of fresh medium basally. Cultivation at the ALI was performed for 5 weeks. Another group of cells, in contrast to the ANaMuc model, were additionally cultivated for 5 weeks under LCC conditions (LiNaMuc model). The cell culture medium was changed three times a week. HuNASA was used as the cell culture medium in all experiments. The MucilAir™ model (Epithelix Sàrl, Plan-les-Ouates, Geneva, Switzerland) consisting of isolated human nasal epithelial cells originating from one healthy donor (Caucasian female, 43 years, batch number MD0748) was used as a reference for the ANaMuc model. For this model, epithelial cells were seeded on a 6.5 mm Transwell^®^ polyester membrane with a 0.4 µm pore size (Costar 3470, Corning, NY, USA) and cultured under LCC conditions for 5 days. On day 6 of the culture, ALI conditions were initiated. On day 39 of the culture, the differentiated models were shipped. In our laboratory, the MucilAir™ tissues were transferred to a 24-well plate filled with 700 µL of prewarmed MucilAir™ culture medium (Epithelix Sàrl, Plan-les-Ouates, Geneva, Switzerland) according to the manufacturer’s instructions. The medium was changed 3 times a week. For the histological characterization of the ANaMuc and LiNaMuc models, the samples were fixed in 4% formaldehyde solution overnight, dehydrated in ethanol, and embedded in Technovit^®^ 7100 (Kulzer GmbH, Hanau, Germany) according to the manufacturer’s specifications. Cross sections were cut to a 4 μm thickness using a microtome (Microm HM 355 S, Thermo Scientific; Waltham, MA, USA) and prepared with HE staining.

### 2.4. Human Nasal Tissue

Tissue specimens of human nasal mucosa were obtained from turbinectomy surgeries in accordance with ethical regulations. All studies included smoking or nonsmoking female and male donors aged between 20 and 70 years. The tissue was placed into DMEM/F12 (PAN Biotech, Aidenbach, Germany) supplemented with 5% FBS, 1% L-glutamine, and 1% antibiotic/antimycotic solution immediately after removal and used for experiments within 2 h.

### 2.5. Immunofluorescence Staining

The isolated primary cells and the immortalized nasal epithelial P1 cell line were analyzed for cell type-specific markers. All samples were washed with PBS and fixed with paraformaldehyde (PFA) for 10 min. Subsequently, the cells were permeabilized with Triton X 0.1% in PBS for 10 min, followed by three washing steps with PBS and blocking with 10% normal goat serum (Vector Laboratories Inc., Burlingame, CA, USA) in PBST (PBS with 0.1% Tween 20) for 45 min at room temperature. The primary antibodies (Table 1) were diluted with 0.1% BSA in TBST (Tris-buffered saline with Tween 20) and incubated overnight at 4 °C. Afterward, the cells were rinsed with PBS three times for 5 min each and incubated with the appropriate secondary antibodies (Table 1), which were diluted in PBS, for 1 h at room temperature. The cell nuclei were counterstained with Hoechst 33342 (Sigma-Aldrich Chemie GmbH, Taufkirchen, Germany) at a ratio of 1:50 in PBS (stock solution 1 mg/mL in 50% ethanol) for 15 min at room temperature and with light protection followed by two washing steps with PBS. All antibodies were imaged using an inverted epifluorescence microscope IX73 (Olympus, Tokyo, Japan) equipped with CellSens imaging software 1.18 (Olympus, Münster, Germany). All antibodies used in this study showed reactivity against human antigens as well as cross-reactivity with porcine antigens.

### 2.6. Alcian Blue Staining

The mucus production of the ANaMuc and LiNaMuc models was checked every week for 5 weeks of ALI and LCC cultivation by detecting glycoproteins in the mucus with alcian blue. Briefly, the cells were washed with PBS and fixed with 4% PFA for 10 min. Then, the cells were rinsed with PBS and 3% acetic acid (*v*/*v*). Subsequently, 1% alcian blue (*v*/*v*) (Sigma-Aldrich Chemie GmbH, Taufkirchen, Germany) was added to the apical chamber of the inserts for 30 min. Afterward, two rinsing steps were performed. Counterstaining of the cell nuclei was performed with nuclear fast red-aluminum sulfate solution (Carl Roth GmbH, Karlsruhe, Germany) for 4 min. Evaluation of the stained samples was performed on a 3D digital microscope (VHX-5000, Keyence, Osaka, Japan). To determine the percentage area covered with mucus, 10 images with a magnification of 400 were taken and spatially analyzed; a hue color filter from 145 to 188 and a saturation filter from 140 to 255 were applied. The settings were evaluated by optical inspection to conservatively extract only blue-stained areas.

### 2.7. Scanning Electron Microscopy (SEM)

The ANaMuc and LiNaMuc model, the MucilAir™ model, and specimens of human nasal mucosa were examined for the occurrence and distribution of ciliated epithelial cells. First, the cells were fixed with a glutaraldehyde solution of 4% in PBS (Carl Roth GmbH, Karlsruhe, Germany) for 90 min, followed by two washing steps with PBS. Subsequently, a 2% (*m*/*v*) osmium tetroxide solution (Carl Roth GmbH, Karlsruhe, Germany) was used to postfix the tissue structures for 2 h. Then, dehydration was performed in an ascending ethanol series with concentrations of 30%, 50%, 70%, and 90% (*v*/*v*) followed by three treatments with absolute ethanol for 30 min each. The samples were finally dried overnight. Prior to measurement, the samples were sputter coated with gold for 140 s at 30 mA in an argon atmosphere (Cressington sputter coater 108 auto, Tescan GmbH, Dortmund, Germany). Finally, the samples were examined by SEM (Phenom XL, Thermo Fisher Scientific, Waltham, MA, USA) using an SE (secondary electron) detector applying a 15 kV electron acceleration voltage at 1 Pa.

### 2.8. TEER Measurement/EIS (Electro-Impedance Spectroscopy)

The barrier properties of the ANaMuc and LiNaMuc models were assessed three times a week by measurements using an Endohm chamber and EVOM^2^ resistance meter (both World Precision Instruments, Sarasota, FL, USA). The raw data were corrected for the baseline resistance of the blank filter. The corrected values were calculated corresponding to the surface area of the inserts (1.12 cm^2^). In addition to the conventional resistance measurement, the impedance and phase shift of the models in the range of 10 Hz to 100 kHz were measured using a potentiostat (Reference 600plus, Gamry Instruments, Warminster, PA, USA). The potentiostat was connected to the Endohm chamber, and the AC excitation voltage was set to 45 mV. To determine the electrical parameters of the electrical impedance spectroscopy (EIS) measurement, an equivalent circuit model was applied, as described by Giampetruzzi et al., and applied in the commercially available cellZscope device (nanoAnalytics, Münster, Germany) [28] (Supplementary Figure S2).

### 2.9. Permeation Experiments

Permeation studies were performed using the ANaMuc model after 21 days of ALI cultivation and using MucilAir™ as a reference. As a marker substance for paracellular permeation, sodium fluorescein (donor concentration 250 µg/mL, Sigma-Aldrich Chemie GmbH, Taufkirchen, Germany) was chosen, rhodamine B (50 µg/mL, Sigma-Aldrich Chemie GmbH, Taufkirchen, Germany) was chosen for transcellular permeation, and FITC-dextran 4000 (mol wt. 3000–5000, Sigma-Aldrich Chemie GmbH, Taufkirchen, Germany) with a concentration of 2500 µg/mL was tested as a marker with high molecular weight. Both models were washed once with prewarmed KRB (Krebs Ringer Buffer) and equilibrated for 30 min in KRB at 37 °C. TEER measurement was then conducted. Afterward, the KRB was removed and replaced on the basolateral side with 2 mL acceptor medium (KRB). On the apical side, 0.4 mL of the test solutions were applied. Due to the cultivation of MucilAir™ on 24-well inserts, the volumes were adjusted in these permeation experiments. The donor volume was 100 µL, and the acceptor volume was 700 µL. Then, 100 µL samples were taken from the acceptor compartment after 10, 20, 30, 60, 90, 120, 150, 210, 270, and 330 min for both models and replaced with the same volume of prewarmed KRB. During the experiment, the plates were shaken horizontally at 150 rpm and 37 °C. All substances were analyzed by fluorescence spectroscopy using a Tecan GENios fluorescence plate reader (Männedorf, Switzerland). An excitation wavelength of 485 nm and emission wavelength of 535 nm were used for sodium fluorescein and FITC-dextran 4000. Experiments with rhodamine B were performed at an excitation wavelength of 535 nm and an emission wavelength of 590 nm. The samples were analyzed, and the permeation coefficient (P_app_) was calculated according to Equation (1):

(1) $$P_{app}=\frac{\mathrm{dQ}}{\mathrm{dt}\cdot c_{0}\cdot \;A}$$

where dQ/dt is the flux (µg/s) of the respective substance across the barrier, c_0_ is the initial donor concentration (µg/mL), and A is the surface area (cm^2^).

### 2.10. Statistical Analysis

The data for the permeation experiments are presented as the mean ± deviation (SD). Statistical analysis of the results was performed using a two-tailed, two-sample F test followed by a two-tailed, two-sample *t* test using SPSS software 28 (IBM, Armonk, NY, USA); *p*-values less than 0.05 (* *p* < 0.05, ** *p* < 0.01, *** *p* < 0.001) were considered to indicate statistical significance.

## 3. Results

### 3.1. Primary Cells

**Figure 1:**
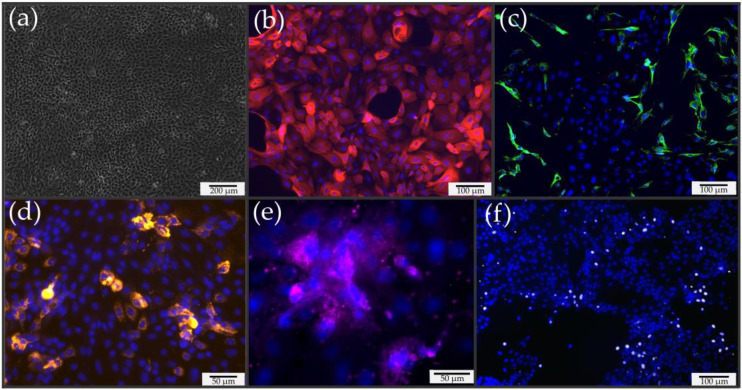
Microscopic image and immunofluorescence staining of porcine primary cells. (**a**) Primary culture after 7 days of cultivation showing formation of a confluent monolayer. (**b**) Detection of nasal epithelial cells via immunofluorescence for pan-cytokeratin shown in red. (**c**) Immunofluorescence staining for vimentin and identification of nasal fibroblasts in primary culture; vimentin-positive cells are shown in green. (**d**) Mucus-forming goblet cells are shown in yellow. Muc5AC served as a specific target. (**e**) Demonstration of a cell cluster with ciliated cells shown in magenta. The detection was performed via gamma-tubulin. (**f**) Immunofluorescence staining for p-63 to detect basal cells. Cells with positive results are shown in white. (**b**–**f**) Cell nuclei stained with Hoechst 33342 are shown in blue.

Both enzymatic isolation with Accutase^®^ and mechanical brushing successfully isolated porcine nasal epithelial cells. The adhesion of the epithelial cells could be observed one day after seeding by both methods. The adhesion of ciliated epithelial cells could only be seen in cell clusters, not in single cells, visibly due to the cilia beat. After 3 days, larger clusters of epithelial cells were already visible, forming a confluent monolayer after 7 days (Figure 1a). The cultivation of primary cells was easily achieved over two more passages. From the third passage, the severely restricted growth and subsequent death of the epithelial cells was observed. Since the isolation of epithelial cells risks introducing fibroblasts into primary cultures, the epithelial cell cultures (Figure 1b) were checked for fibroblast contamination by staining for vimentin (Figure 1c). To generate pure epithelial cell lines, the proportion of fibroblasts should be kept as low as possible. Using these methods, the proportion of vimentin-positive cells was approximately 10–20%. In previous studies, the number of fibroblasts was lower in cultures obtained using the brush method than in those obtained by enzymatic digestion using Accutase^®^ enzymes. In addition, primary cultures were examined for specific cell types (Figure 1d–f). Goblet cells were detected with an anti-Muc5AC antibody, basal cells were detected with an anti-p63 antibody, and ciliated epithelial cells were labeled with an anti-gamma tubulin antibody. All cell types of the nasal epithelium could be detected with the preparation methods used.

### 3.2. Immortalized Cell Line P1

**Figure 2:**
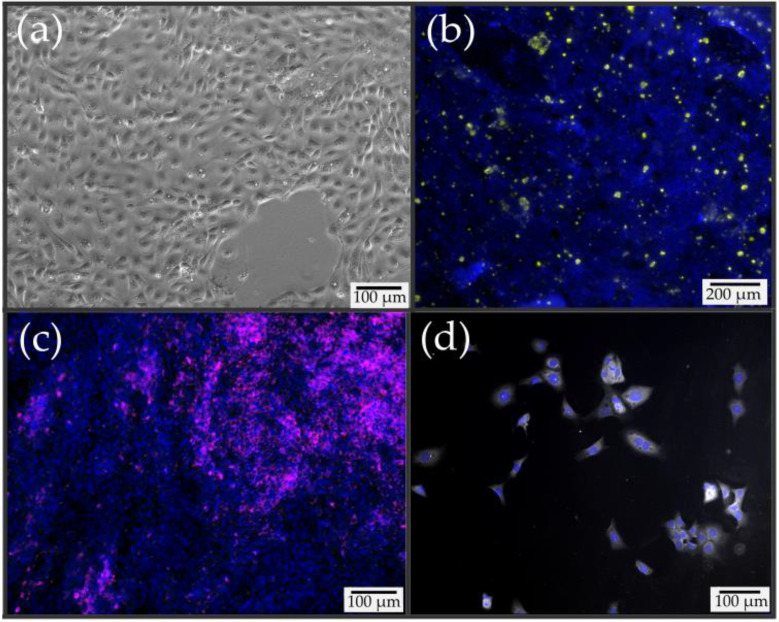
Microscopic image and immunofluorescence staining of the immortalized nasal epithelial P1 cell line. (**a**) Light microscope image 3 days after seeding. (**b**) Yellow indicates mucus formed by goblet cells, which show a homogeneous distribution in the cell layer after ALI cultivation. Muc5AC served as a specific target of the antibodies. (**c**) Demonstration of ciliated epithelial cells in multiple areas of the cell layer during ALI cultivation visualized in magenta. The detection was performed via gamma-tubulin. (**d**) Detection of basal cells via immunofluorescence for KRT5. KRT5-positive basal cells are shown in white. Detection was performed during LCC cultivation by immunofluorescence. (**b**–**d**) Cell nuclei stained with Hoechst 33342 are shown in blue.

Based on the primary cultures, a new cell line was established with a novel cell expansion approach [27], which is detailed in the Section 2. The P1 cell line showed good and fast adhesion to collagen-coated substrates and high proliferation capacity. Figure 2a shows the typical appearance of the P1 cell line under light microscopy. The phenotype remained stable over at least 30 passages. Figure 2 also shows the presence of goblet cells, ciliated epithelial cells, and basal cells detected by immunofluorescence staining. Goblet cells and ciliated epithelial cells (Figure 2b,c) were found among the cells that underwent ALI cultivation; basal cells were also identified during cell proliferation (Figure 2d). Thus, the P1 cell line has the ability to form every cell type occurring in the nasal epithelium. In addition, the ability of this cell line to form a paracellular barrier very quickly was demonstrated via the detection of ZO-1 (Supplementary Figure S1).

### 3.3. Cell Culture Models

**Figure 3:**
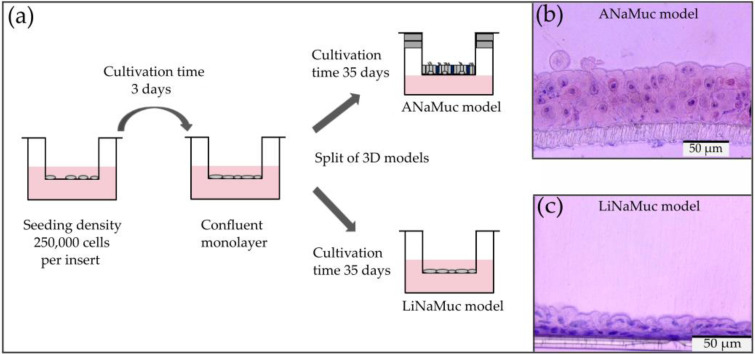
(**a**) Culture procedure of the different 3D cell culture models. (**b**) ANaMuc model, HE staining after 35 days of ALI cultivation. (**c**) LiNaMuc model, HE staining after 35 days of LCC cultivation.

Figure 3a shows the procedure for culturing the different 3D models. The ANaMuc model was cultured for 5 weeks, and the influence of the air stimulus on the expression of typical markers of nasal epithelial cells was investigated. For this purpose, comparisons were made with the LiNaMuc model that was cultured exclusively under LCC conditions for 5 weeks. A fundamental difference in the histology of the immortalized P1 cell line was observed between cultivation under LCC exclusively and cultivation at the ALI after reaching confluence. Cultivation under liquid resulted in the development of small, rather round cells that did not show directional growth. The height of the cell layer was approximately 25 µm (Figure 3c). The ANaMuc model showed a pseudostratified organization with heights up to 100 μm (Figure 3b). Similar heights of the epithelial cell layer had already been found in human tissue sections [17,29]. Cells of different shapes could be observed within the model. Near the membrane, the cells were predominantly small and round, similar to the typical shape of basal cells, while elongated cells were observed extending above this layer to the cell surface.

### 3.4. Alcian Blue Staining

**Figure 4:**
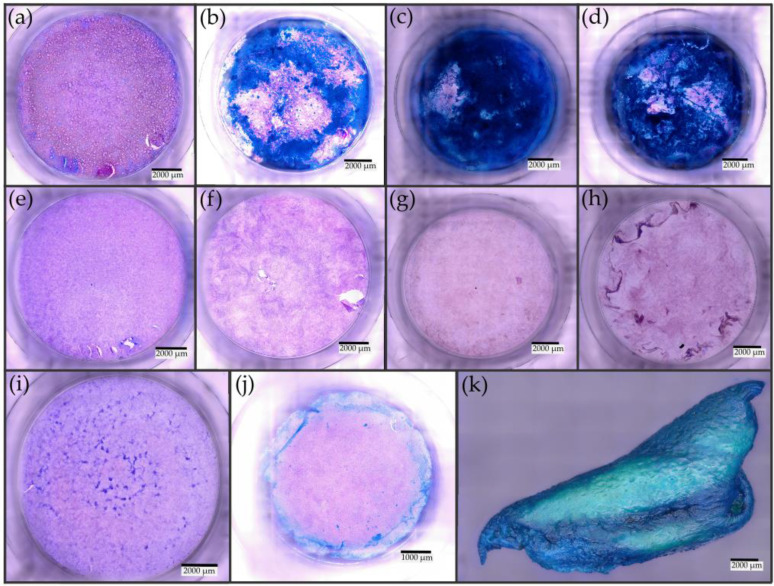
Top view of alcian blue staining in cell culture inserts of ANaMuc and LiNaMuc models compared with MucilAir™ and excised human nasal mucosa tissue. (**a**–**d**) ANaMuc model with alcian blue staining. (**a**) After 7 days; the first accumulations of mucus can be seen in the marginal areas. (**b**) After 21 days; a significant increase in the mucus-covered cell surface compared with 7 days of ALI was observed. (**c**) After 28 days; almost the entire cell layer shows coverage with mucus. (**d**) After 35 days. (**e**–**h**) LiNaMuc model with alcian blue staining. (**e**) After 7 days. (**f**) After 21 days; in contrast to the ANaMuc model, no increase in mucus was detected. (**g**) After 28 days. (**h**) After 35 days. (**i**) Alcian blue staining after reaching confluence of the P1 cell line; subsequently, the samples were separated into ALI and LCC cultivation. (**j**) The MucilAir™ tissue construct only showed significant accumulations of mucus in the marginal area. (**k**) Native human nasal mucosa showed complete coverage of the epithelial cell layer with mucus.

The effect of the ALI cultivation method (ANaMuc model) on the formation of mucus versus complete culturing under LCC conditions (LiNaMuc model) was examined over a 5-week period with alcian blue staining. The results of this investigation are shown in Figure 4. The findings for both models were compared with those for MucilAir™ and nasal mucosa of human origin. First, staining was performed before the cells were separated into the different cultivation procedures. The percentage of mucus detected on the cell surface was 4.5% (Figure 4i). After 7 days, no major differences were observed between the ANaMuc and LiNaMuc models (Figure 4a,e). An increase in the detected mucus area could be observed between the 7th and 14th day of ALI-cultivated cells from 2.8% to 22.3%. In the following weeks, the mucus-covered area of the ANaMuc model continued to increase steadily, reaching a maximum of 93% on day 28 of ALI cultivation (Figure 4c). In contrast, no increase in the area covered by mucus compared to the baseline value was observed in cells undergoing continued LCC cultivation. Indeed, the areas stained by alcian blue even showed a decrease from day 21 of LCC cultivation compared to the baseline value. Thus, the significant position of ALI cultivation in comparison with submerged cultivation was demonstrated for the P1 cell line. In comparison with the established MucilAir™ tissue construct, the ANaMuc model showed clear advantages regarding mucus production. After 28 days of ALI cultivation, almost the entire cell surface of this model was covered with mucus, and the microscopic image of MucilAir™ only showed significant accumulations of mucus in the marginal area. The remaining cell layer was covered only by fine dot-like staining. The analysis of the microscopic images revealed a stained area of 11.7% (Figure 4j). In parallel to mucus staining of the tissue constructs, control staining was performed on native human mucosa specimens (Figure 4k). Strongly pronounced and uniform staining of the epithelial surface was demonstrated. These results are consistent with reports from previous studies [29,30].

### 3.5. Scanning Electron Microscopy (SEM)

**Figure 5:**
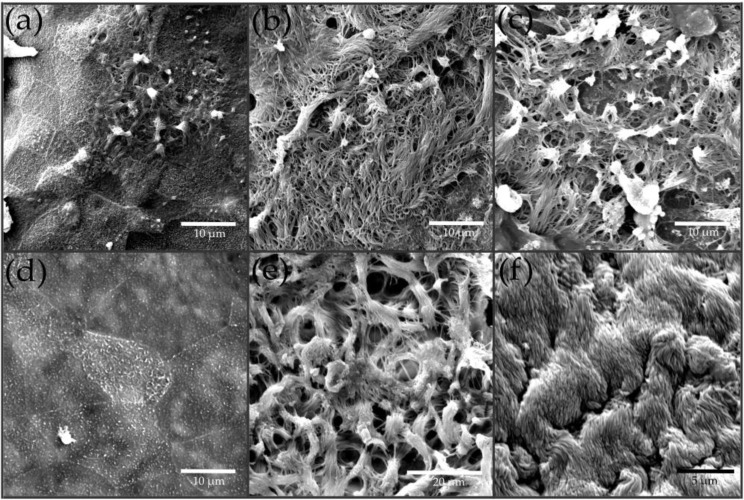
Scanning electron micrographs (SEM) of the ANaMuc and LiNaMuc models compared with MucilAir™ and excised human nasal mucosa tissue. (**a**–**c**) ANaMuc model after 7, 21, and 35 days of ALI. (**a**) Development of the first cilia after 7 days. (**b**) Models after 21 days showing a significant increase in ciliated cells; larger areas with dense cilia are interrupted by areas without cilia. (**c**) After 35 days, mature cilia with homogeneous distribution over a large area of the cell layer were detected; smaller areas of cells with microvilli appeared between cells with fully formed cilia. (**d**) LiNaMuc model after 35 days of LCC cultivation showing an absence of mature cilia. (**e**) The MucilAir™ tissue construct showed strong formation of mature cilia with uniform distribution throughout the cell layer. (**f**) Native human nasal mucosa showed comparable distribution and density of detected cilia to MucilAir™.

Figure 5 presents the results of SEM examination of the ANaMuc and LiNaMuc models with respect to the formation and distribution of ciliated epithelial cells at different cultivation time points. The results were compared with MucilAir™ and excised human nasal mucosa tissue. The ANaMuc model showed the first ciliated cells after 7 days at the ALI. The formation occurred in smaller cell clusters at different locations of the cell layer (Figure 5a). An increase in the number of ciliated cells was observed with the continuation of this cultivation method over time. After 35 days of ALI cultivation, cilia were visible with a homogeneous distribution over a large area on the cell layer (Figure 5c). Smaller areas of cells with microvilli appeared between cells with fully formed cilia. In comparison with the MucilAir™ model and human nasal mucosa tissue samples, both showing nearly complete coverage of the cell layer with cilia (Figure 5e,f), the porcine tissue construct was yet not able to fully represent in vivo conditions.

The importance of air as a stimulus for the formation of cilia is particularly evident by comparing samples of the LiNaMuc model exclusively cultured under submerged conditions with samples of the ANaMuc model after 35 days of ALI cultivation. While the P1 cell line is able to grow cilia on the cell surface under air, no formation of cilia was observed under LCC cultivation during the entire 35-day period (Figure 5d). Only partial microvilli could be detected.

### 3.6. TEER Measurement/EIS (Electro-Impedance Spectroscopy)

**Figure 6:**
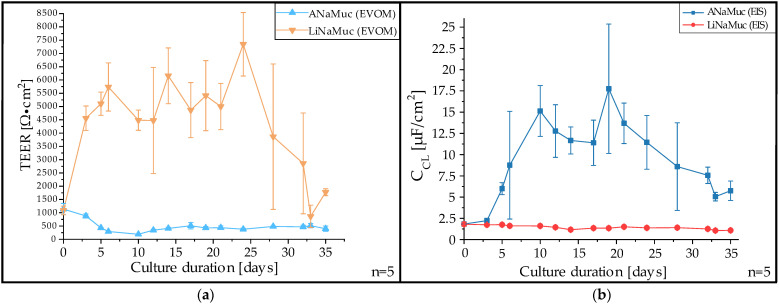
Determination of the transepithelial electrical resistance (TEER) and capacitance (C_CL_) of the ANaMuc and LiNaMuc models. (**a**) TEER values measured by an EVOM resistance meter. The TEER values of the ANaMuc model are more comparable to in vivo conditions than the values determined for the LiNaMuc model. (**b**) Cell layer capacitance determined from EIS measurement for the ANaMuc and LiNaMuc models. Mean ± SD, *n* = 5.

The effect of the different cultivation modes of the LiNaMuc and ANaMuc models on the expression of barrier properties was investigated and compared using TEER measurements. TEER values were measured by both the EVOM resistance meter and the Reference 600plus; the values at 12.5 Hz were subsequently compared. Data obtained from both methods showed a high level of agreement (Supplementary Figure S2). In Figure 6a, the TEER values measured by the EVOM resistance meter are plotted against the culture duration up to 35 days. A clear distinction between the LiNaMuc and ANaMuc models could be demonstrated by significant differences in the recorded TEER values.

Before separation into the different cultivation methods (day 0), TEER values of 930–1470 Ω cm^2^ were measured. With further LCC cultivation, the TEER value increased sharply and reached a mean value of 4580 Ω cm^2^ on day 3 after separation of the groups. Over the following 3 weeks, the mean value of the LCC samples never fell below 4000 Ω cm^2^. In the last days of cultivation, there was a decrease in TEER; however, the measured values never fell below the TEER range measured for ALI cultivation. With the start of ALI cultivation, a reduction in TEER was observed. Up to the 10th day, when a mean value of 190 Ω cm^2^ was measured, a steady decrease in TEER was detected. Subsequently, a slight increase in the strength of the paracellular barrier was recorded, with TEER values falling within a constant range between 340 and 510 Ω cm^2^ until the last day of cultivation.

In addition to the conventional resistance measurement, the impedance and phase shift of the models in the range of 10 Hz to 100 kHz were measured using Reference 600plus. Figure 6b shows the change in the cell layer capacitance over the observed cultivation duration. In contrast to the decreased TEER values, the ANaMuc model showed a substantial increase in capacitance from 1.9 µF/cm^2^ to 15 µF/cm^2^ within the first 10 days. After a peak value of 17.5 µF/cm^2^ was reached on the 19th day, the capacitance began to decline slightly. In contrast, the capacitance of the LiNaMuc model remained constant between 1.5 and 2 µF/cm^2^ throughout the cultivation.

### 3.7. Permeation Experiments

**Figure 7:**
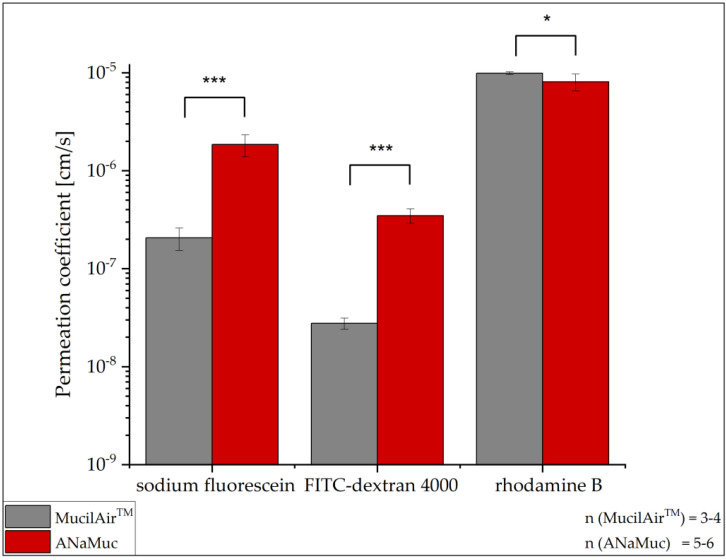
Permeation studies on the ANaMuc model and MucilAir™ using sodium fluorescein, FITC-dextran 4000, and rhodamine B as marker substances for molecules with varying physicochemical properties. Mean ± SD, *n* = 3–6, * *p* < 0.05, *** *p* < 0.001.

In addition to evaluating the barrier function of the 3D models of the P1 cell line via TEER measurement, comparative permeation studies of the ANaMuc model and MucilAir™ were performed using sodium fluorescein, FITC-dextran 4000, and rhodamine B. The permeation coefficients of both investigated models were calculated and are shown in Figure 7 and summarized in Table 2. The MucilAir™ model showed a significantly lower permeation coefficient than ANaMuc for the marker substances with hydrophilic properties (sodium fluorescein) and high molecular weight (FITC-dextran 4000). The P_app_ values of rhodamine B were approx. 8.1 ± 1.6 · 10^−6^ cm/s for the ANaMuc model and 9.8 ± 0.3 ∙ 10^−6^ cm/s for MucilAir™, indicating that both models had very similar permeability to the lipophilic marker rhodamine B in contrast to their permeabilities to sodium fluorescein and FITC-dextran 4000.

## 4. Discussion

Two different isolation methods were used to obtain primary cultures of porcine nasal epithelial cells in this study: epithelial cells were obtained by direct smear (brush), and the cells were separated enzymatically via the use of Accutase^®^. In general, isolated cells obtained from both preparation methods adhered, but due to the ciliary beat, single ciliated epithelial cells could not adhere to the growth substrate. This observation is consistent with the findings of Million et al. [35]. The adhered cells grew rapidly and could be subcultured until the 3rd passage. The limited cultivation time of three to four passages has also been described previously [13,36]. With regard to the purity of the obtained epithelial cells and the detection of individual nasal mucosa cell types, there were no apparent significant advantage of either of the isolation methods.

The P1 cell line derived from the immortalization process of porcine nasal primary cells was tested for suitability as a new model. In contrast to previously immortalized nasal epithelial cells [37,38], all cell types of the nasal epithelium were detected via immunofluorescence with specific markers; therefore, this important prerequisite for further investigations of this cell line was fulfilled. The P1 cell line was then used for the construction of new 3D models that were studied in more detail. The main focus was to closely mimic the in vivo characteristics of the nasal mucosa. For this purpose, the influence of air on the differentiation of the new cell line was investigated in comparison to cultivation under liquid. While cultivation of the 3D models under liquid (LiNaMuc model) did not result in a satisfactory morphology of the cell layer, cultivation at the ALI (ANaMuc model) resulted in a morphology similar to the morphology of in vivo cells. Thus, polarized elongated cells could be seen above the small round cells near the PET membrane in the ANaMuc model.

Following the morphological characterization of the new ANaMuc model, the requirements for mucociliary clearance were examined. Cultivation under the ALI resulted in a steady increase in the detected mucus areas on the cell layer up to almost complete coverage, whereas no increase in mucus was observed for the LiNaMuc model. The increased mucus production in cultivation conditions that more closely mimicked in vivo conditions was consistent with observations already reported for human nasal primary cells [8,39,40,41]. In contrast to the ANaMuc model, significant deviations were observed for the MucilAir™ model. The area of the cell layer covered by mucus was smaller, and significant accumulation was only visible in the marginal areas of the inserts. Nevertheless, the adequate production of mucus has already been demonstrated for MucilAir™ [42,43]. In addition, the different cell culture media and the different cultivation protocols for the ALI must also be considered in this comparison.

In addition to the production of mucus, the formation of motile cilia represents another prerequisite for mucociliary clearance. While no cilia were observed for the LiNaMuc model during the entire cultivation period of 35 days, cilia expression was found for the ANaMuc model. The behavior of the immortalized cell line corresponded to observations of human nasal epithelial cells, for which cultivation under the ALI was also required for the formation of cilia [12,41,44]. For the ANaMuc model, the expression of motile cilia was successfully demonstrated after 7 days at the ALI. The incipient formation of cilia after a comparably short duration of ALI cultivation has also been observed in human nasal primary cells [12,40]. At this time point, ciliated cells only appeared in small cell clusters. The length of most cilia at this time point was also different compared to studies of later time points. In the following weeks, a significant increase in the size of cell clusters with ciliated epithelial cells was observed. However, a lower cilia density was observed on the cell surface in a direct comparison of the ANaMuc model after 35 days at the ALI with control samples of human nasal mucosa and MucilAir™ tissue constructs. One possible reason for the difference in cilia expression between ANaMuc and MucilAir™ could be due to different cultivation protocols. For the ANaMuc model, the cells were seeded directly onto collagen-coated inserts. Differentiation was subsequently induced by the air stimulus, and the cell culture medium, used over a period of 35 days. Wiszniewski et al. described a technique to increase the proliferation and differentiation of human airway epithelial cells using an airway fibroblast feeder layer; they proved that, after 30 days, the number of ciliated epithelial cells was approximately 15 times higher when epithelial cells were cultured in the presence of fibroblasts than when they were cultured without fibroblasts [45]. Very long cultivation periods of 5 months increased the number of ciliated epithelial cells up to 90%. Additional studies of ANaMuc constructs over a period longer than 35 days could provide information on whether the number of cilia on the cell surface can be further increased.

In view of its potential use as a new model to study drug permeation, the barrier properties of the ANaMuc models were first investigated via regular measurement of TEER. While very high TEER values, which were not comparable with the in vivo situation, were measured over the entire cultivation period for the LiNaMuc models, a reduction in TEER was observed for the ANaMuc models in the first days of cultivation. After approximately 10 days of ALI cultivation, a stable TEER range was reached, which was between 340 and 510 Ω cm^2^. Thus, TEER values were demonstrated for the ANaMuc model that were closer to in vivo values than those previous measured in human nasal primary cells in culture [22,46]. A possible reason for the decreasing TEER values during ALI cultivation could be the stimulated differentiation of P1 cell line due to air cultivation. The expression of tight junctions between goblet cells and neighboring cells is less pronounced than that between neighboring ciliated epithelial cells [47]. The electron microscopic analysis of samples obtained by the freeze-fracture technique revealed that the tight junctions in the area of goblet cells are discontinuous and fragmented [48]. Thus, goblet cells represent weak points in the epithelial association and reduce the strength of the paracellular barrier. Clear differences between ANaMuc and LiNaMuc were also found regarding cell layer capacitance. The capacitance of the LiNaMuc models showed no significant changes during the entire cultivation, whereas clear changes could be detected for the ANaMuc models. The boost in capacitance during the first days of ALI cultivation also corresponds to the appearance of cilia and goblet cells, as evidenced by the expanded secretion of mucus. As van der Helm et al. showed for the differentiation of gut epithelial cells, an increase in the cell layer capacitance was observed in accordance with the appearance of villi and microvilli [49]. Furthermore, a decreasing capacitance was determined by the development of a multilayered cell structure of a reconstructed epidermis [50]. Thus, the steady decrease in the capacitance of the ANaMuc model after day 19 occurred simultaneously with the pseudostratified organization of the cells and the growing film of mucus covering the cell layer.

To further verify the suitability of the ANaMuc model, permeation tests with three different model substances were performed, and the obtained permeation coefficients were compared with the reference MucilAir™. Table 2 summarizes the permeation coefficients of ANaMuc and MucilAir™ to sodium fluorescein and FITC-dextran 4000 and provides an overview of the comparative values previously reported in the literature. Compared with the permeability of porcine and excised human tissue as well as RPMI 2650 cells, the ANaMuc model showed higher agreement with respect to the permeation properties of sodium fluorescein than MucilAir™. In contrast, human nasal primary cells examined for permeability by Agu et al. showed significantly lower values in a range of 0.045–0.191 10^−6^ cm/s in comparison with the ANaMuc model [51]. However, these values are very close to the permeation coefficients of sodium fluorescein determined for MucilAir™ in our study. Contrary to our study, Mercier et al. measured a significantly larger permeation coefficient of approx. 5 10^−6^ cm/s for MucilAir™ [42]. One possible reason for the different results could be the lower TEER values (316 ± 31 Ω cm^2^) in their study compared to our measurement (433 ± 43 Ω cm^2^). However, TEER values in the range of 400–650 Ω cm^2^ are not uncommon for MucilAir™, as shown in studies by Furubayashi and Welch, which reported values of 560 ± 34 Ω cm^2^ and 346 ± 14 to 638 ± 81 Ω cm^2^, respectively [52,53]. A significantly higher permeation coefficient was also determined for FITC-dextran 4000 in the ANaMuc model in comparison to MucilAir™ (Table 2). However, the results obtained with ANaMuc again showed high agreement with excised nasal mucosa from sheep and rabbit as well as the optimized RPMI model previously described. Thus, the permeability of the ANaMuc model to hydrophilic and high molecular weight substances determined in our study was closer to those of excised nasal mucosa tissues than the permeability measured for MucilAir™. The reason for this effect is the lower strength of the paracellular barriers of the ANaMuc model and native tissue, which were detected via TEER measurement, since both substances preferentially permeate via the paracellular pathway.

## 5. Conclusions

Using CI SCREEN technology, the immortalized P1 cell line was obtained from isolated porcine nasal epithelial cells. The suitability of this new cell line was confirmed by detecting all cell types typically found in the nasal epithelium via immunofluorescence. Our results demonstrated that the newly introduced ANaMuc model features both an intense production of mucus and the formation of a satisfactory number of cilia after culturing at the ALI for a period of 35 days. The epithelial integrity, as determined by TEER measurements, showed a high similarity to excised human turbinates. In addition, the ANaMuc model appears to be more similar to excised nasal mucosa in terms of permeability than MucilAir™. Due to the promising results achieved in this study using the static ANaMuc model, further investigations have been carried out under dynamic conditions using a microfluidic chip system, which are the subject of a subsequent publication (part 2). However, future studies will be directed toward establishing an immortalized cell line of human origin to further increase the degree of transferability of results from such an in vitro system to the human in vivo conditions.

## Figures and Tables

**Table 1:** List of antibodies used in this study.

Antibody	Antigen	Purpose	Host	Dilution	Supplier, Reference
Anti-pan Cytokeratin (C11)	Cytokeratin peptides 4,5,6,8,10,13,18	Detection of epithelial cells	Mouse	1:400	Abcam (Boston, MA, USA), ab 7753
Anti-Vimentin (VI-10)	Vimentin	Evaluation of contamination	Mouse	1:1000	Abcam, ab 20346
Anti-Mucin5AC (45M1)	Peptide core of Muc5AC	Goblet cell marker	Mouse	1:1000	Abcam, ab 212636
Anti-gamma Tubulin (TU-30)	Gamma Tubulin	Detection of ciliated cells	Mouse	1:500	Abcam, ab 27074
Anti-p63 (4A4)	N-terminus of ΔN p63 isoform of mouse protein	Basal cell marker	Mouse	1:100	Abcam, ab 735
Anti-Cytokeratin 5	Cytokeratin 5	Basal cell marker	Rabbit	1:500	Abcam, ab 53121
Anti-Mouse IgG H&L Alexa Fluor 568	Whole molecule mouse IgG	Secondary antibody	Goat	1:400	Abcam, ab 175473
Anti-Rabbit IgG H&L FITC ^1^	Whole molecule rabbit IgG	Secondary antibody	Goat	1:200	Abcam, ab 97079

^1^ FITC: fluorescein isothiocyanate.

**Table 2:** Summary of permeation coefficients (10^−6^ cm/s) of sodium fluorescein and FITC-dextran 4000 for the ANaMuc model and MucilAir™. Mean ± SD, *n* = 3–6.

Nasal Mucosa Model	Sodium Fluorescein	FITC-Dextran 4000
ANaMuc	1.851 ± 0.469	0.513 ± 0.076
MucilAir™	0.20 ± 0.056	0.028 ± 0.004
RPMI 2650	2.04 ± 0.1 ^a^	0.69 ± 0.04 ^a^
Excised nasal mucosa	3.12 ± 1.99 (human) ^b^	0.46 (sheep) ^d^
	3.61 ± 3.53 (porcine) ^c^	0.52 -1.275 (rabbit) ^e^

Ref. ^a^ [16], ^b^ [15], ^c^ [31], ^d^ [32], ^e^ [33,34]. Comparison with P_app_ values of the same marker compounds determined using animal or human ex vivo models and nasal epithelial cells (RPMI 2650) reported in the literature.

## Data Availability

Data are available upon reasonable request from the corresponding author.

## Supplementary Materials

The following supporting information can be downloaded at https://www.mdpi.com/article/10.3390/pharmaceutics15092245/s1, Figure S1: Immunocytochemical detection of tight junction protein ZO-1. Figure S2: TEER data obtained for ANaMuc and LiNaMuc model by electro-impedance spectrometry (EIS) and single-frequency (SF) measurement.
