# Supplementary material for: The Path from Nasal Tissue to Nasal Mucosa on Chip: Part 1—Establishing a Nasal In Vitro Model for Drug Delivery Testing Based on a Novel Cell Line
Source: Pharmaceutics. 2023 Aug 30;15(9):2245. doi: 10.3390/pharmaceutics15092245 (PMC10536430; doi:10.3390/pharmaceutics15092245)
Supplement: Supplementary file 1 [file pharmaceutics-15-02245-s001.zip › pharmaceutics-2486759-supplementary.pdf]

Figure S1

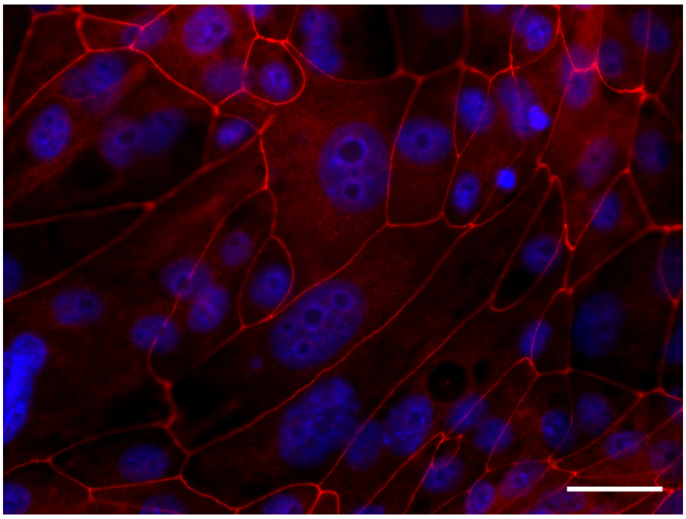

Figure S1. Immunocytochemical detection of the tight junction protein ZO-1 in a confluent monolayer of the P1 cell line. The bar corresponds to 10 µm.

Figure S2

Equivalent circuit model

The proposed equivalent circuit, shown in Figure S2, consists of two equally contributing electrodes, expressed by the constant phase elements (CPE) and the resistance of the media (R_{Med}) in the apical and basal compartments. The cell layer is represented by the ohmic resistance (TEER), reflecting the transport properties through the cell layer, and a parallel capacitance, providing additional information about the properties of the cell layer.

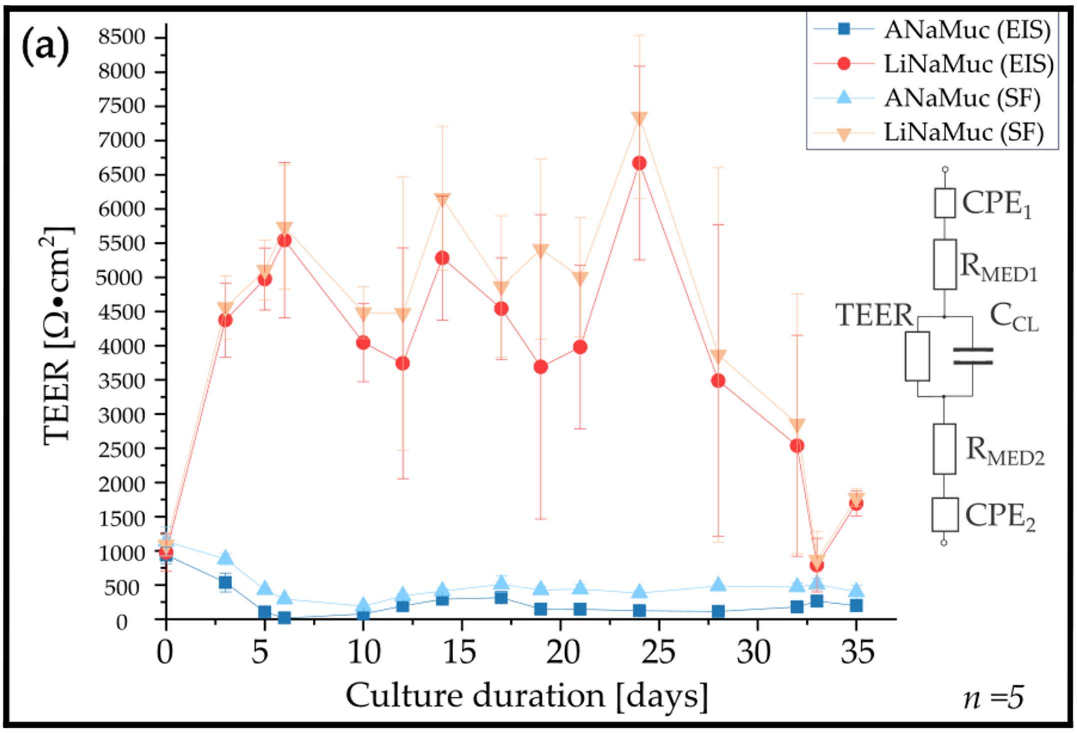

Figure S2. Determination of the transepithelial electrical resistance (TEER) for the ANaMuc and LiNaMuc models by electroimpedance spectrometry (EIS) and single frequency (SF) measurement using an EVOM² resistance meter at 12.5 Hz. TEER values of the ANaMuc and LiNaMuc models were monitored throughout the 35-day cultivation

period using both methods. The characteristic values of the EIS measurement were obtained from the equivalent circuit model on the right-hand side in Figure S2. Mean ± SD, n=5.

Comparison of calculated TEER values

TEER values were measured by both the EVOM² resistance meter (single frequency-SF) and the Reference 600plus (Electro Impedance Spectroscopy-EIS); then, their values at 12.5 Hz were compared. Data obtained from both methods showed a high level of agreement. Before separation into the different cultivation methods (day 0), values of 930–1470 $\Omega \cdot cm^2$ were measured (SF). Values in the range of 880–1300 $\Omega \cdot cm^2$ were measured by EIS. With further LCC cultivation, the TEER value increased sharply and reached a mean value of 4580 $\Omega \cdot cm^2$ (SF) on day 3 after separation of the groups. At this time, the mean EIS measurement was approximately 4250 $\Omega \cdot cm^2$. In the following 3 weeks, the mean value of the considered samples never fell below 4000 $\Omega \cdot cm^2$ (SF) or 3500 $\Omega \cdot cm^2$ (EIS). In the last days of cultivation, there was a decrease in TEER. However, the measured values never fell below the TEER range measured for ALI cultivation. With the start of ALI cultivation, a reduction in TEER was observed. Up to the 10th day of ALI cultivation, when a mean value of 190 $\Omega \cdot cm^2$ (SF) was determined, a steady decrease in TEER was detected. The decrease in TEER could also be seen in the EIS measurement. On the 6th day of cultivation, the lowest barrier property was determined to be approx. 20 $\Omega \cdot cm^2$. Subsequently, a slight increase in the strength of the paracellular barrier was recorded, with a constant TEER range of 340–510 $\Omega \cdot cm^2$ (SF) measured until the last day of cultivation. The EIS measurement showed values between 100 $\Omega \cdot cm^2$ and 280 $\Omega \cdot cm^2$ for this period.
